# Evolutionary divergence of embryo implantation in primates

**DOI:** 10.1098/rstb.2021.0256

**Published:** 2022-12-05

**Authors:** Dylan Siriwardena, Thorsten E. Boroviak

**Affiliations:** ^1^ Department of Physiology, Development and Neuroscience, University of Cambridge, Downing Site, Cambridge CB2 3EG, UK; ^2^ Centre for Trophoblast Research, University of Cambridge, Downing Site, Cambridge CB2 3EG, UK; ^3^ Wellcome Trust – Medical Research Council Stem Cell Institute, University of Cambridge, Jeffrey Cheah Biomedical Centre, Puddicombe Way, Cambridge CB2 0AW, UK

**Keywords:** primate, embryo implantation, human development, trophoblast, primate embryo

## Abstract

Implantation of the conceptus into the uterus is absolutely essential for successful embryo development. In humans, our understanding of this process has remained rudimentary owing to the inaccessibility of early implantation stages. Non-human primates recapitulate many aspects of human embryo development and provide crucial insights into trophoblast development, uterine receptivity and embryo invasion. Moreover, primate species exhibit a variety of implantation strategies and differ in embryo invasion depths. This review examines conservation and divergence of the key processes required for embryo implantation in different primates and in comparison with the canonical rodent model. We discuss trophectoderm compartmentalization, endometrial remodelling and embryo adhesion and invasion. Finally, we propose that studying the mechanism controlling invasion depth between different primate species may provide new insights and treatment strategies for placentation disorders in humans.

This article is part of the theme issue ‘Extraembryonic tissues: exploring concepts, definitions and functions across the animal kingdom’.

## 1. Introduction

Embryo implantation defects are a major cause of pregnancy failure in humans [1,2]. Implantation is mediated by trophectoderm, the outer layer of the preimplantation embryo, which subsequently differentiates to form the fetal portion of the placenta. Errors in trophoblast development and invasion have been linked to numerous pregnancy complications, including miscarriage, pre-term labour, pre-eclampsia and placenta accreta spectrum disorders [3–5]. Complications can also persist after birth, with neurological or growth syndromes originating from defective placental development [6–8].

Human embryo implantation has remained elusive owing to the inaccessibility of early implantation stages. Most of our anatomical knowledge about this process has been sourced from classic histological sections of the Boyd and Carnegie collections [9,10] in tandem with preimplantation and, more recently, postimplantation *in vitro* embryo culture. However, preimplantation studies miss the crucial initial attachment of the embryo and *in vitro* postimplantation cultures lack the complex three-dimensional multicellular environment of the uterus. Therefore, our current understanding of early primate embryo implantation is largely derived from model organisms such as rodents (mouse and rat), New World monkeys (common marmoset (*Callithrix jacchus*)), Old World monkeys (rhesus macaque (*Macaca mulatta*), cynomolgus macaque (*Macaca fascicularis*) and baboon (*Papio* sp.)), lesser apes (agile gibbon (*Hylobates agilis*)) and great apes (chimpanzee (*Pan troglodytes*)).

**Figure 1.**
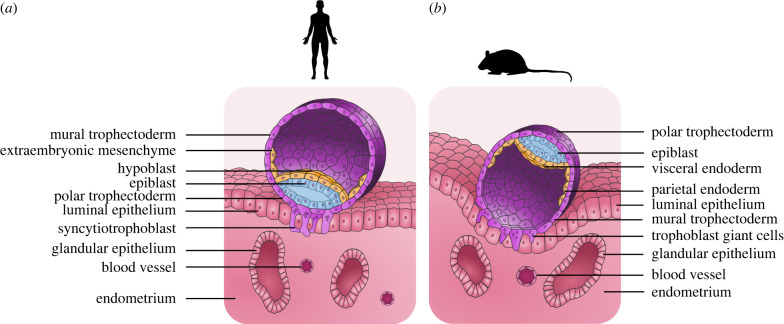
Early blastocyst adhesion and invasion in primate and mouse. Diagram depicting the different cell types during blastocyst implantation in human and mouse. (*a*) Polar trophectoderm in human differentiates into syncytiotrophoblast, which invades into the luminal epithelium. (*b*) Mural trophectoderm in mouse differentiates into trophoblast giant cells.

Key transcriptional regulators of human trophectoderm specification and development were first identified in mouse, including *Cdx2*, *Tead4* and *Gcm1* [11–16]. In both mice and humans, trophectoderm formation and uterine receptivity regulate implantation via cyclic hormones and embryo–maternal crosstalk [17–22]. However, primate implantation differs from rodent with regard to embryo orientation, the cell types mediating implantation, and the lineage potential of early trophoblast cells. Mouse blastocysts implant with the mural side of the embryo (the trophectoderm compartment away from the inner cell mass (ICM)) and generate invasive multinucleated trophoblast giant cells [23] (figure 1). The polar side of the embryo (the trophectoderm adjacent to the ICM) remains proliferative and forms extraembryonic ectoderm that expands and differentiates into the labyrinthine structure of the mouse placenta. By contrast, primate embryos first attach with the polar side, wherein polar trophectoderm differentiates into multinucleated primary syncytiotrophoblast and proliferative cytotrophoblast. The invasive primary syncytiotrophoblast penetrates the basal lamina of the luminal epithelium and is essential for the formation of fluid-filled lacunae to facilitate histotrophic nutrition of the embryo [24–26].

In this review, we systematically compare trophoblast development and the early stages of embryo implantation in human and non-human primates. We discuss the steps leading up to implantation in both, the embryo and the uterus, the interactions between the conceptus and maternal tissues, as well as the initial stages of embryo attachment. Finally, we highlight how the evolutionary divergence between individual primate species can provide an avenue to elucidate trophoblast invasion, which will further our understanding of the pathophysiology of implantation and related pregnancy disorders.

## 2. Primates exhibit a wide range of implantation and placentation strategies

**Figure 2.**
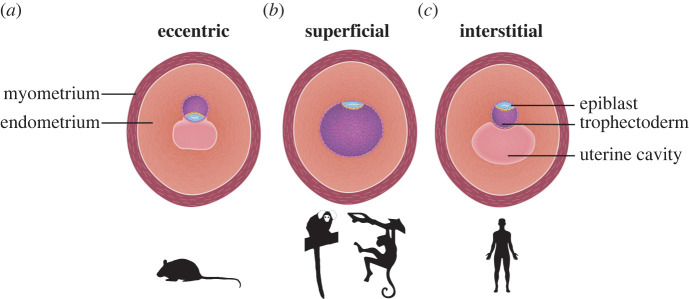
Types of implantation. Types of implantation strategies employed by mouse, marmoset, rhesus macaque and human. (*a*) Eccentric implantation: mouse. (*b*) Superficial implantation: marmoset, rhesus macaque, baboon and cynomolgus macaque. (*c*) Interstitial implantation: chimpanzee and human.

There is considerable variation in implantation type, invasion depth, maternal remodelling, and placentation between primates (table 1) [27]. Implantation type refers to the location of the embryo with regard to the uterine lining, and can be categorized as superficial, eccentric or interstitial (figure 2). In superficial implantation, the embryo remains within the uterine cavity (lemur, marmoset, baboon, rhesus macaque) and is often associated with shallow trophoblast invasion [27]. With eccentric implantation (mouse, rat) the embryo partially embeds into the uterine tissues, leaving portions of the conceptus exposed to the uterine cavity. Interstitial implantation entails the embryo penetrating deep into the uterus and becoming fully engulfed in the endometrial tissue (lesser apes, great apes). Interestingly, interstitial implantation can be found in a variety of species, including guinea pigs, bats and humans. Owing to their large phylogenetic differences, it is likely the evolution of interstitial implantation evolved independently in these organisms [28].

**Table 1.** Summary of implantation strategies in primates. Mens.: menstruation, Decid.: decidualization, BM: luminal basement membrane.

Common name	Sub-order, Family	Species	Primate group	Implantation and Placentation type	Interhaemal membrane	Pattern	Mens.	Decid.
slender loris	Strepsirrhini, Lorisidae	*Loris tardigradus*	lemurs and lorises, *Strepsirrhini*	superficial, diffuse	epitheliochorial, no BM breach	villous	N	at implantation
mouse lemur	Strepsirrhini, Cheirogaleidae	*Microcebus murinus*	lemurs and lorises, Strepsirrhini	superficial, diffuse and discoid	epitheliochorial and endotheliochorial, no BM breach	villous	N	at implantation
common marmoset	Haplorhini, Callitrichidae	*Callithrix jacchus*	New World monkeys, Ceboidea	superficial, bidiscoid	haemochorial, BM breach	villous	N	at implantation
rhesus macaque	Haplorhini, Cercopithecidae	*Macaca mulatta*	Old World monkeys, Cercopithecidae	superficial, bidiscoid	haemochorial, BM breach	villous	Y	at implantation
cynomolgus macaque	Haplorhini, Cercopithecidae	*Macaca fascicularis*	Old World monkeys, Cercopithecidae	superficial, bidiscoid	haemochorial, BM breach	villous	Y	at implantation
baboon	Haplorhini, Cercopithecidae	*Papio*sp.	Old World monkeys, Cercopithecidae	superficial, discoid	haemochorial, BM breach	villous	Y	at implantation
western gorilla	Haplorhini, Hominidae	*Gorilla gorilla*	great apes, Hominidae	interstitial, discoid	haemochorial, BM breach	villous	Y	pre-decidualization, small amount
chimpanzee	Haplorhini, Hominidae	*Pan troglodytes*	great apes, Hominidae	interstitial, discoid	haemochorial, BM breach	villous	Y	pre-decidualization
human	Haplorhini, Hominidae	*Homo sapiens*	great apes, Hominidae	interstitial, discoid	haemochorial, BM breach	villous	Y	pre-decidualization

Equally, the shape and organization of the placenta can vary considerably between primate species. Lemurs and lorises (*Strepsirhini*) develop a diffuse placenta that covers the, entire surface of the uterine luminal epithelium and is invaginated with villi [29,30]. By contrast, ‘dry-nosed’ primates (*Haplorhini*) have a discoid placenta covering a smaller circular area [30]. Many New World and Old World monkeys form a bidiscoid structure with two circular placentae on opposing ends of the uterine cavity [27]. Lesser and great apes develop a single discoid placenta. Most primates give rise to villous placentae, wherein the functional units are chorionic villi, which are often organized into tree-shaped structures [30–32].

Further variation can be found in the number of tissue layers between the fetal and maternal circulation, which often correlates with invasion depth. Several species of lemur exhibit extremely shallow invasion and thus employ epitheliochorial or endotheliochorial placentation (table 1) [27]. In epitheliochorial placentation, the luminal epithelium is maintained throughout gestation [33]. Endotheliochorial placentation invades more deeply, breaching the luminal epithelium but retaining the maternal endothelial cells surrounding blood vessels [34,35]. Most primates, including New World, Old World and apes, invade even more deeply and undergo haemochorial placentation (humans, marmosets, rhesus macaques, baboons) [35–38]. Haemochorial placentation breaches both the luminal epithelium and endothelial cells, enabling fetal tissues to come into direct contact with maternal blood [35–38].

Studying the morphological, physiological and molecular features of placentation in different primate species can help us to identify conserved processes applicable to all primates. Conversely, the differences in implantation and placentation might be used to dissect human-specific processes. Knowing the mechanisms controlling trophoblast invasion depth in primates of different implantation modes may inform new treatments for placental disorders resulting from either too shallow (pre-eclampsia) or too deep (placenta accrete spectrum) trophoblast invasion.

## 3. Trophectoderm compartmentalization prepares the embryo for implantation

In primates, trophoblast is specified in the first lineage decision at the 16–32 cell stage. Inhibition of Hippo signalling in the outer blastomeres induces trophectoderm specification, while the inner blastomeres are directed toward an ICM fate [12,39]. Trophectoderm cells proliferate and cavitate to form the blastocyst. Subsequently, the trophectoderm compartmentalizes into the polar trophectoderm, adjacent to the ICM, and mural trophectoderm on the opposite side, encompassing the blastocoel. At embryonic days 6–7, the human blastocyst hatches from the zona pellucida, and within the next 3–5 days implants into the luminal epithelium. Trophectoderm mediates implantation in four stages: (i) trophectoderm–uterine crosstalk, (ii) apposition, (iii) adhesion and (iv) primary invasion. The initial contact between the trophectoderm and the luminal epithelium is established by signalling crosstalk. During apposition, the implantation site and embryo orientation are decided via loose connections between the trophectoderm and the luminal epithelium. Adhesion occurs as the initial connections become tighter, enabling invasive trophoblast subtypes to push past the luminal epithelium and establish access to maternal histotrophic nutrition [25,26,40].

Trophectoderm compartmentalization into polar and mural trophectoderm is essential for proper implantation [41]. In mice, polar trophectoderm is enriched for *Cdx2* and *Esrrb,* while transcripts for *Ascl2*, *Ndrg1*, *Krt18, Tfap2c* localize towards the mural side [13,42,43]. Human blastocysts exhibit regionalized expression of FGFR1 and CCR7 in the polar trophectoderm [44,45], and single-cell transcriptome profiling revealed co-expression of *GATA2*, *GATA3*, *CDX2* and *KRT18* in a subcluster of trophectoderm [11,44]. Trophectoderm subpopulations with increased levels of *CDX2* have also been observed in cynomolgus macaques and marmosets [46–48]; however, in the absence of spatial information, it is unclear whether these cells truly represent polar trophectoderm. A recent study revealed enrichment of *NR2F2* in polar trophectoderm of human blastocysts [49], but *NR2F2* expression spread throughout the entire trophectoderm within 2 days [49]. It is currently unclear whether this spread is specific to *NR2F2* alone, if polar trophectoderm cells proliferate into the mural compartment, or if the polar phenotype is adopted by the entire trophectoderm as it matures. Notably, in rhesus macaques and marmosets, the mural trophectoderm implants at the opposite side of the uterine cavity (bidiscoid) after the initial polar implantation [38,46,50]. This mode of implantation would be in line with the hypothesis that the mural trophectoderm gradually adopts a polar trophectoderm phenotype in the implanting blastocyst.

Human and non-human primate embryo implantation is initiated at the polar end, as opposed to mouse, which implants at the mural side (figure 1). Microvilli, which appear at the late blastocyst stage, mediate the initial interactions between polar trophectoderm and the luminal epithelium. [24,38]. In both rodents and primates, implanting trophectoderm differentiates to form invasive cells that attach and penetrate the luminal epithelium (figure 1). In mouse, the mural trophectoderm differentiates into multinucleated giant cells. In primates, the polar trophectoderm gives rise to multinucleated cells during primary syncytiotrophoblast formation [51]. This is facilitated by the human luminal epithelium, which becomes more apoptotic [52] and secretes factors to promote primary syncytiotrophoblast in both mural and polar trophectoderm [53].

Primary syncytiotrophoblast breaches the basal lamina of the luminal epithelium and invades the uterine lining, thus ensuring strong attachment to maternal tissues. It is important to note that primary syncytiotrophoblast differs from the secondary syncytiotrophoblast that covers the surface of villi in the human placenta, which can erode surrounding tissue but has little invasive character. While the exact timing of primary syncytium formation remains unclear, we know that primary syncytiotrophoblast forms in human embryo postimplantation *in vitro* cultures in the absence of maternal tissues [54–56]. In line with this observation, rhesus macaque and baboon-hatched blastocysts also establish binucleated cells, which are localized within the polar trophectoderm [38,57]. Preimplantation trophectoderm expresses fusion proteins, including *ERVV2*, which may poise trophectoderm for primary syncytiotrophoblast differentiation even before embryo adhesion [55,56,58]. Further studies will be required to determine how embryo adhesion controls and promotes primary syncytialization.

## 4. Maternal remodelling regulates uterine receptivity

The preparations for successful embryo implantation begin long before fertilization. Cycles of pituitary and ovarian hormones regulate periodic changes in uterine receptivity and therefore the ‘window of implantation’.

The follicular, or proliferative, phase of the oestrus cycle promotes proliferation of uterine endometrium via high oestrogen and follicle-stimulating hormone levels [59–62]. During this phase, stromal cells proliferate and differentiate to expand the uterine lining [63]. This is followed by the luteal, or secretory, phase, where higher progesterone levels sustain the uterine endometrium for embryo implantation [59,61]. As progesterone levels decline, the uterine lining is either reabsorbed in rodents, strepsirrhines and some New World monkeys, including the marmoset, or shed in menstruation in humans, greater and lesser apes, Old World monkeys and some New World monkeys, including the tufted capuchin [64,65]. The importance of progesterone signalling is underlined by the fact that progesterone inhibitors effectively prevent embryo implantation in rodents and primates [19,66–68].

Progesterone induces decidualization of stromal cells in the endometrium. Decidualization refers to the transformation of stromal cells into larger, polyhedral decidual cells [69–72]. In chimpanzees, gorillas and humans, decidualization occurs before implantation [73], which has been linked to the evolution of interstitial implantation. In other primates, decidualization only occurs after implantation. Consequently, evolutionary analysis of decidualization prior to implantation represents an avenue to understand the requirements for human implantation [64].

In all primates, decidualization is sustained after embryo implantation and during trophoblast invasion [72,74–77]. Primary syncytiotrophoblast secretes chorionic gonadotropin (CG), which preserves the corpus luteum. This is of pivotal importance to sustaining high progesterone levels, which in turn prevents menstruation and loss of the implanted embryo [78]. The luminal epithelium further supports implantation by absorbing intrauterine fluid, thus promoting the narrowing of the uterine cavity [52,79]. Intrauterine volume is used as a contraindication of fertility in IVF procedures in humans [79].

Immune cells equally play a profound role in regulating uterine receptivity, decidualization and invasion. During the secretory phase, natural killer (NK) cells, dendritic cells, lymphocytes and macrophages are recruited to the uterine lining [71,72]. NK cells and macrophages are enriched at the implantation site in rhesus macaques [80], and decreases in NK cells at implantation sites have been associated with miscarriage in baboon [81]. In humans, reduced NK cell numbers and activity equally correlate with implantation failure [82,83]. The evolution of implantation has been linked to the development of anti-inflammatory mechanisms and decidualization [84,85], emphasizing the importance of studying the immune response and decidualization in non-human primates.

In most Old World and New World monkeys, including rhesus macaques, baboons and marmosets, the luminal and glandular epithelia remodel to form large, glycogen-rich cells termed epithelial plaques [38,50,76]. Epithelial plaque formation radiates out from the implantation site and increases during the first 10 days of pregnancy [38,46,86]. In marmosets, the entire luminal epithelium remodels into epithelial plaques, including regions in the periphery of the embryonic compartment itself [46]. It has been suggested that epithelial plaques are similar to decidualized cells, but the precise role of epithelial plaques in uterine receptivity and implantation remains elusive.

## 5. Embryo–maternal crosstalk prior to embryo implantation

**Figure 3.**
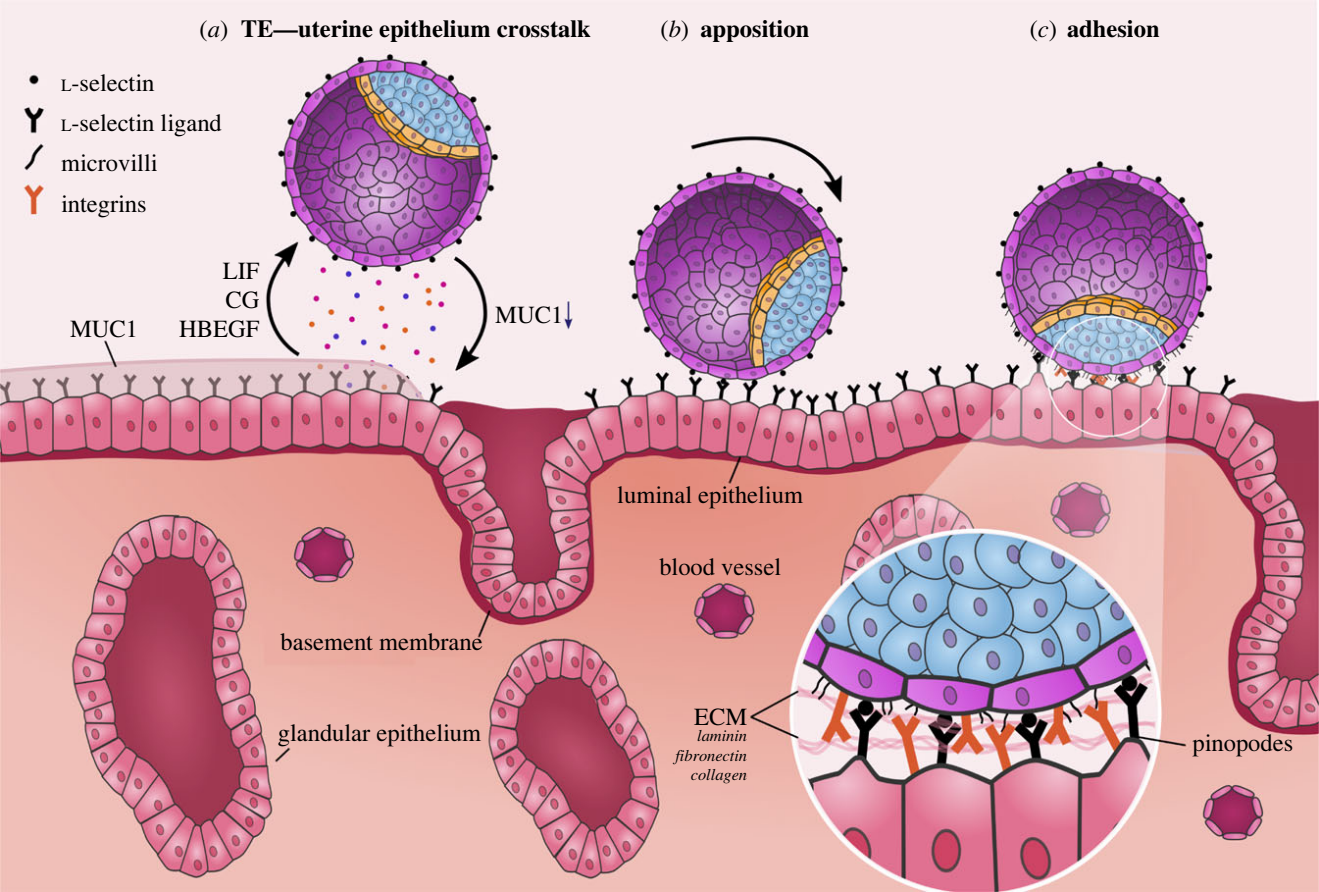
Apposition and adhesion of blastocyst to luminal epithelium. (*a*) Secreted ligands signal between the trophectoderm (TE) and luminal epithelium, downregulating MUC1 in the luminal epithelium and exposing uterine ECM and adhesion proteins. (*b*) Transient interactions between l-selectin and l-selectin ligands slow embryo rolling and establishes initial adhesion. (*c*) Integrins on the TE and luminal epithelium bind to apically secreted ECM from both cell types.

The trophectoderm and luminal epithelium secrete a wide array of ligands preceding embryo implantation to adjust the outer layer of the blastocyst for adhesion and invasion (figure 3) [87–90]. Concomitantly, embryo secretions promote uterine receptivity and decidualization [91–93].

Heparin-binding EGF-like growth factor (HBEGF) is a member of the EGF protein family and binds several receptors including EGFR, ERBB2 and ERBB4 [94]. HBEGF is secreted by human endometrial epithelia and maximally expressed in the luteal (or secretory) phase of the oestrus cycle [95]. HBEGF binds to ERBB4, which is expressed in human trophectoderm [95] and improves blastocyst development *in vitro* [96]. Human blastocyst culture experiments with HBEGF-coated slides showed increased adhesion of the embryos to the surface [97]. Moreover, EGFR and ERBB4 binding promotes syncytiotrophoblast formation [87–89]. In rhesus macaque, trophoblast motility and proliferation was increased in EGF-treated *in vitro* cultured embryos [88]. Collectively, this suggests that HBEGF signalling plays an important role in trophoblast development and priming the embryo for attachment and invasion.

Leukaemia inhibitory factor (LIF) is a cytokine from the interleukin (IL)6 family and an essential regulator of the early embryo and pluripotent stem cells [42,98–100]. LIF is essential for the establishment of a successful pregnancy in humans and rhesus macaques [101–103]. During implantation, LIF reinforces its own expression by upregulating other inflammatory agonists such as IL1, IL6 and TNFα in the endometrium [69,91,104]. Inflammatory cytokines, including IL1, induce CG expression in the human embryo [105] and integrin β3 expression in endometrium [106]. Human CG both activates and inhibits LIF, through IL1 and IL6, respectively [92], suggesting dynamic regulation of LIF signalling via trophectoderm–uterine crosstalk during implantation.

The human embryo resides in the uterine cavity for approximately 72 h prior to implantation [107]. It is tempting to speculate that mechanisms exist to prevent premature implantation. Mucins coat the apical surface of the luminal epithelium and have been suggested to inhibit implantation in both rodents and primates [108–112]. Mucins are large glycoproteins in mucosal barriers that are capable of steric receptor inhibition between the embryo and maternal tissues [113,114]. In mouse, Muc1 is globally reduced during the luteal phase [115]. Both, Muc1 knockout and the enzymatic removal of Muc1 increase embryo receptivity [110]. Primate MUC1 is upregulated in the luminal epithelium of the baboon and human uterus during the early luteal phase [116–118]. In the tufted capuchin, MUC1 coats the oviduct but is reduced in the luminal epithelium, potentially indicating a role in preventing ectopic pregnancy [109]. Both rhesus macaque and baboons lose MUC1 in the entire luminal epithelium prior to trophoblast attachment [119]. Interestingly, the embryo itself seems to regulates the luminal epithelium in human, as blastocyst implantation assays *in vitro* showed local reduction of MUC1 in endometrial cultures around the embryo [118]. Nevertheless, future studies are required to functionally interrogate whether the luminal epithelium influences, or even determines, the prospective implantation site.

## 6. Transient interactions orient the embryo during apposition

Apposition refers to the initial interactions between the embryo and the endometrial lining of the uterus. In this process, transient connections between the trophectoderm and the luminal epithelium establish the final orientation of the implanting embryo (figure 3). In mouse, the first interactions occur between the mural side of elongated E4.5 embryos and both sides of the uterine cavity, essentially enclosing the embryo in an upright position [23]. It is postulated that the flattened mural trophectoderm becomes more adhesive and attaches to the luminal epithelium, which then bulges around the elongated E4.5 embryo to orient the embryo correctly [23]. However, in primates, the blastocyst does not undergo asymmetrical elongation, nor does the trophectoderm contact both uterine cavity walls simultaneously. Electron microscopy in rhesus macaque revealed interactions outside the polar trophectoderm that result in polar orientation, with the ICM toward the endometrium [38]. This raises the question of how primate blastocysts orient correctly for implantation.

l-selectin (*SELL*) is a type-I transmembrane glycoprotein with well-established roles in circulating leucocytes [120]. l-selectin binds to a variety of ligands broadly grouped as sialylated and fucosylated carbohydrate molecules, which can be found on mucin-like glycoprotein membrane receptors [121]. Mouse *SELL* knockout embryos still implant, suggesting that these interactions are not essential for embryo adhesion [122]. In humans, l-selectin is expressed on trophoblast [123], while l-selectin ligands are expressed on the luminal epithelium during the luteal phase [124]. Interestingly, in leucocytes and neutrophils, l-selectin aids in their ‘rolling action’ in blood vessels [120,125], which bears resemblance to the rolling embryo [124,126]. This may suggest that selectins promote transient interactions to slow the embryo, allowing other embryo–uterine interactions to occur within the local area (figure 3).

Trophinin (*TRO*) is an apical membrane protein that has been implicated in mediating implantation [127–130]. In humans, trophinin is expressed by both maternal and trophoblast cells at the implantation site in normal and ectopic pregnancies [130–133]. Trophinin-binding induces EGF signalling in human trophoblast stem cells, which promotes syncytiotrophoblast formation [88]. The embryonic pole of the blastocyst is enriched for trophinin in the rhesus macaque [134], and *TRO* is lowly expressed in marmoset trophectoderm [46]. Therefore, trophinin-binding may assist in embryo orientation toward the polar trophectoderm by inducing the formation of invasive primary syncytium.

## 7. Integrin-binding mediates stable adhesion

After apposition, stronger adhesions between the polar trophectoderm and the luminal epithelium are established via integrin binding [135–137] (figure 3). Integrins are transmembrane receptors composed of α and β subunits that mediate cell–extracellular matrix (ECM) adhesion [138,139]. In mouse, human and baboon, α and β integrins are upregulated in both the luminal epithelium and TE during the luteal phase [137,140–144]. Integrin α5β3, α3β3, α4β6 and αvβ5 are expressed in the human luminal epithelium and have been suggested to play a role in blastocyst attachment [18,135,144–150] Integrins bind a variety of ECM components, including fibronectin, vitronectin, laminin and collagen IV [151–153]. Laminin and collagen IV expression is increased at the implantation site and throughout the endometrium [144], while apical fibronectin increases in human and mouse blastocysts [154,155]. At implantation, both integrins of the implanting trophectoderm and integrins of maternal luminal epithelium attach to apically presented ECM molecules. After attachment, integrin-binding remains important and promotes trophoblast invasion past the luminal epithelium in multiple species, including mouse, rhesus macaque, marmoset and human [139,150,156–158]. Collectively, the transient surface protein binding during apposition [159] is followed by more stable interactions via integrins on the polar trophectoderm and luminal epithelium, both of which bind to apical ECM.

## 8. Primary syncytium breaks through the uterine lining

**Figure 4.**
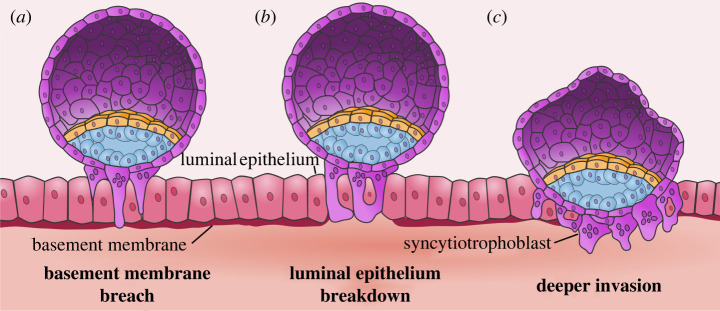
Initial invasion of luminal epithelium. Invasion of multinucleated syncytiotrophoblast past the luminal epithelium. (*a*) Syncytiotrophoblast projections push between uterine epithelia and breach the basement membrane. (*b*) Syncytiotrophoblast envelops the uterine epithelia. (*c*) Luminal epithelium is degraded as deeper invasion occurs.

The initial embryo invasion process in primates consists of three main steps: (i) penetration of syncytium between luminal epithelium, (ii) breach of the uterine basal lamina, and (iii) breakdown of uterine epithelial cells (figure 4). Histological sections from implanting embryos in rhesus macaque, marmoset and baboon reveal multinucleated cells penetrating the luminal epithelium [38,57,76]. Cytoplasmic protrusions push between and surrounding epithelial cells during early implantation in rhesus macaque and baboon [37,38,57,76]. After projecting between the luminal epithelium, syncytiotrophoblast begins to break down the uterine basal lamina. Marmoset and rhesus macaque syncytium expresses matrix metalloproteinases (MMPs), which aid in breaking down the basal lamina [46,160,161]. Syncytium projections in lemurs breach the basal lamina but do not envelop the luminal epithelium (figure 4) [37]. Consequently, the epitheliochorial mode of placentation found in lemurs provides an opportunity to identify specific regulators of luminal epithelium breaching. By contrast, during marmoset, rhesus macaque, and baboon embryo implantation, the luminal epithelium at the implantation site is broken down by a multinucleated syncytium that completely surrounds luminal epithelial cells and destroys them (figure 4). Interestingly, in sheep, binucleated cells invade and incorporate engulfed maternal epithelial cells [162,163]. It remains unclear whether similar luminal epithelium–syncytium fusions occur in primates [164] or if the maternal nuclei are broken down [165]. In mouse, the entire luminal epithelium undergoes apoptosis after implantation, including regions away from the implantation site [166,167]. By contrast to mouse, primates sustain a distinct layer of cells lining the uterine cavity [38,50,57].

Ultimately, the embryo is surrounded by maternal tissues. Human and chimpanzee embryos quickly become surrounded by the decidualized endometrium, almost appearing to move into the endometrium [10,168]. Human stromal cells migrate in response to trophoblast cells [169]. This suggests the intriguing possibility that the embryo is encapsulated by migratory stromal cells, rather than actively burrowing into the maternal tissues [170].

## 9. Differences in implantation and invasion depth between primate species as a platform to study trophoblast invasion

**Figure 5.**
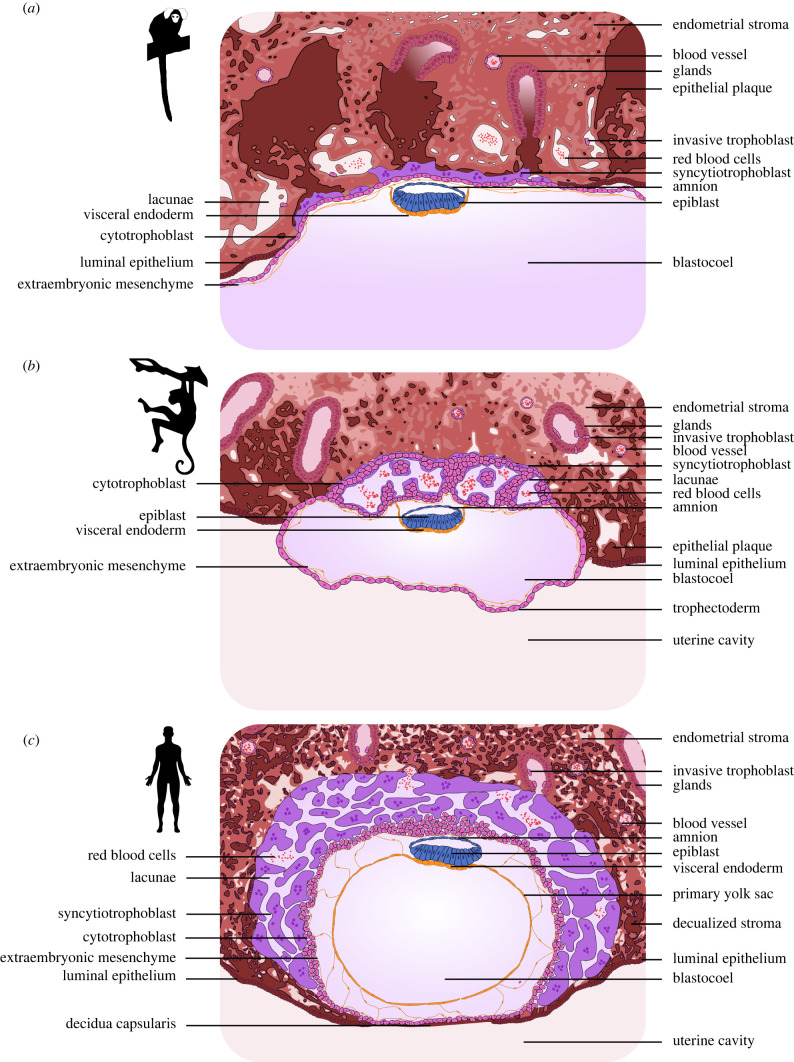
Implantation depth in primates at lacunar stage. Lacunar stage in (*a*) New World monkeys (marmoset), (*b*) Old World monkeys (rhesus macaque and baboon) and (*c*) great apes (human).

Primate species display considerable variation in embryo implantation and invasion depth (figure 5). In New World and Old World monkeys, which undergo superficial implantation, trophoblast invades to a lesser extent into the endometrium than in great apes, where the embryo implants interstitially [37]. Even among superficially implanting primates, the trophoblast invades to a variable degree. In marmoset, the trophoblast remains close to the uterine basal membrane and forms a thin layer of syncytiotrophoblast [24,46,50]. By contrast, primary syncytium invades substantially deeper into the maternal tissues in rhesus macaque and baboon [38,57,76].

Cross-species analysis of trophoblasts from species with varying invasion depths could reveal important regulators of invasion. Indeed, there is significant overlap in placental genes associated with superficial implantation and placental disorders characterized by too shallow trophoblast invasion, such as pre-eclampsia. Candidate regulators for increased invasion depth and pre-eclamptic placentae included *VGLL1, FLT1*, *CD97* and *EGLN3* [171]. *VGLL1* is expressed in human cytotrophoblast as well as invasive extravillous trophoblast [172], and *FLT1* is a *VEGF* receptor that promotes extravillous trophoblast motility [173–175]. Thus, comparing the mechanisms of superficial implantation and invasion in postimplantation *in vivo* samples will provide promising candidates for functional studies to illuminate the pathophysiology of pre-eclampsia in the future.

Extravillous trophoblast is specified from cytotrophoblast shortly after embryo implantation and consists of highly migratory cells that rapidly invade the endometrium. Extravillous trophoblast cells play an important role in immune regulation, promote decidualization and modulate the maternal vasculature during early pregnancy [176,177]. In particular, extravillous trophoblast remodels the spiral arteries to protect early placental structures from high-pressure blood and to ensure proper perfusion of the developing placenta [2,178].

Rhesus macaque, baboon and marmoset, all of which undergo superficial implantation, vary greatly in extravillous trophoblast abundance and trophoblast invasion depth [27]. Marmosets generate minimal extravillous trophoblast, only surrounding arteries near the placental zone [24]. By contrast, both rhesus macaque and baboon exhibit deep extravillous trophoblast invasion [31,36]. In baboons, extravillous trophoblast surrounds spiral arteries as early as embryonic day 13 [31]. Human and chimpanzee spiral arteries undergo the most extensive remodelling [179]. Future cross-species analysis of the recently generated *in vivo* single-cell transcriptome datasets of postimplantation trophoblast in human, rhesus macaque and marmoset will facilitate the discovery of new regulators of extravillous trophoblast differentiation [46,48,58].

## 10. Conclusion

Trophectoderm specification, embryo orientation, adhesion and invasion are all essential for proper embryo development. As early human implantation stages are not accessible, non-human primate models are imperative for our understanding of embryo implantation. The evolutionary divergence in implantation strategies among primate species presents an exciting opportunity to elucidate early trophoblast invasion by studying candidate regulator activities across primate species with varying invasion depth. The results from this research will be important to delineate the molecular mechanisms underlying pathophysiological changes in human placental development.

## Data Availability

This article has no additional data.
